# Team interventions in acute hospital contexts: protocol for the evaluation of an initial programme theory using realist methods

**DOI:** 10.12688/hrbopenres.13225.1

**Published:** 2021-03-29

**Authors:** Una Cunningham, Aoife De Brún, Mayumi Willgerodt, Erin Blakeney, Eilish McAuliffe

**Affiliations:** 1 Centre for Interdisciplinary Research, Education and Innovation in Health Systems (UCD IRIS), School of Nursing, Midwifery & Health Systems, University College Dublin, Belfield, Dublin 4, D04 V1W8, Ireland; 2 Pillar Centre for Transformative Healthcare, Mater Misericordiae University Hospital, Eccles St, Dublin 7, D07 R2WY, Ireland; 3 School of Nursing, Department of Biobehavioral Nursing and Health Informatics, University of Washington, Seattle, WA, 98195, USA

**Keywords:** Multi-disciplinary, Team, Quality, Safety, Realist, Evaluation, Theory, Hospital, Context

## Abstract

**Introduction**: Literature on multi-disciplinary healthcare team interventions to improve quality and safety of care in acute hospital contexts tends to focus on evaluating the success of the intervention by assessing patient outcomes. In contrast, there is little focus on the team who delivered the intervention, how the team worked to deliver the intervention or the context in which it was delivered. In practice, there is therefore a poor understanding of why some interventions work and are sustained and why others fail. There is little emphasis in the literature on how the team delivering the intervention might impact success or failure.

Given that team is the vehicle through which these interventions are introduced, it is important to understand interventions from their perspectives.

This research seeks to deepen understanding of enablers and barriers for effective team interventions. Using two case studies, we will evaluate previously developed initial programme theories to understand, what worked for whom, in what conditions, why, to what extent and how?

**Methods and analysis**: A realist evaluation approach will be employed to test the previously formed set of initial programme theories. Two multi-disciplinary acute hospital team interventions in two different geographical and organisational contexts will be identified. In case study 1, a theory based approach to interviewing will be used. In case study 2, interview transcripts obtained using a semi- structured approach for primary research purposes will undergo secondary analysis.

This will enable a more sensitive look at patterns and variations in patterns of multi-disciplinary team interventions. Researchers will first iteratively interrogate each respective dataset to identify the characteristics or resources present within the specific context that influenced how the team intervention worked to produce particular outcomes. Data will then be synthesised across contexts in order to produce middle range theories and thereby more generalisable insights.

## Introduction

In acute hospital contexts, the primary purpose of most multi-disciplinary teams (MDTs) is to provide quality patient care in a co-ordinated way as patients require interventions from across several clinical areas and functions. Choi and Pak ^1^ describe MDTs as teams "where disciplines operate within their own boundaries" (p.225). Hospital staff may, however, identify with several multi-disciplinary teams including those that provide other functions; for example, governance and leadership, service or quality improvement, innovation or re-design and project or change management functions.

Given staff turnover in hospitals, and the dynamic system that requires teams to form and re-form, MDTs tend to be construed as multi-dimensional constructs with fluctuating team structures and processes depending on team composition, team purpose and other clinical teams and functions with whom they interact ^2^. As a result, teams have many inter-dependencies and operate within uncertain conditions. Many are formed on the need for professional role representation and membership may be fluid and *ad hoc* ^3– 6^. By their nature, MDTs are therefore complex and operate within complex dynamic open systems ^7^ that reflect matrix management structures and hierarchical professional structures.

West and Lyubovnilova ^8^ caution that in some instances, it is difficult to define multi-disciplinary healthcare teams , i.e., they do not meet traditional definitions of what constitutes a ‘team’ and refer to their operation as “pseudo-like groups” (p. 8). These teams are further complicated by their composition of multiple healthcare professionals each having their own identity, culture, educational background and objectives. Consequently, professional boundaries, status and power differences will affect interprofessional collaboration [8]. These factors may help to explain why healthcare literature often lacks specificity in descriptors of multi-disciplinary teams involved in interventions to improve quality and safety of care ^8, 9^.

Teamwork failures are increasingly cited as significantly impacting on patient safety with concomitant costs to patients, hospitals and consequently the wider economy ^10– 12^.

There has been a sizeable growth in the area of implementation science, and the planning and implementation of team interventions to support the delivery of high quality and safe patient care ^13, 14^. Over the past decade in particular, team interventions have attracted increased research attention in comparison to the previous decade with an emphasis on interventions to improve quality and safety in areas including acute care ^15^; emergency departments ^16^ maternity units ^17^, intensive care units ^18^ and trauma units ^19^.

Whilst numerous studies address interventions by teams to improve quality and safety of care in hospitals ^20– 24^, there is still a dearth of high quality evidence on interventions to improve team effectiveness ^13^.

Emphasis to date has largely been on whether effective teams yield positive results for patients or whether team interventions work or not to produce specific outcomes. In contrast, little attention is given in the literature to the team delivering the intervention and the context in which it is being implemented e.g. detail of team composition, team dynamics, team communication or organisational supports. As MDTs are the vehicle for improvement in quality and safety, understanding how and why team members engage with innovation and improvement interventions is important for both their implementation ^25^ and adaptation to open systems or ‘real world’ contexts ^26^. It is recognised that there is a dynamic interplay between team intervention and context ^27^. It is therefore necessary to understand contextual details of team interventions in order to understand the mechanisms that influence the outcomes of interventions. The dearth of such research, however, means there is little understanding of how and why the team itself impacts on the delivery of these interventions and their success or failure.

For the purpose of this research, team interventions have been defined as:

*An intervention where a team of two or more disciplines is trying to improve how the team delivers patient care- for example: quality improvement, service improvement or change initiatives; process re-design or team training events* ^28^.

As illustrated in their systematic review, Buljac- Samardzic *et al.* categorise team interventions into four primary categories: training tools, organisational re-design and programmes or a combination of these three ^14^. These interventions tend to be multi-layered and complex as teams involved in their introduction are affected by cultural, leadership, financial and other organisational factors making them highly variable and context dependent ^29^. Exploring team interventions and their effectiveness without appropriate consideration of context therefore seems meaningless. Attendance to the interplay between contextual factors and aspects of the intervention could illuminate why and how an intervention may be more impactful in one setting compared to another and should constitute valuable learning for intervention designers and for researchers. 

Each hospital context has a uniqueness and a specific workplace culture and therefore its own specific requirements to support change and improvement ^30^. Identification of patterns in these unique settings that can subsequently be extrapolated to general principles should help to guide implementation of multi-disciplinary team interventions in hospitals. Understanding the conditions under which teams tend to enact certain types of co-ordination mechanisms is critical to creating the conditions for effective performance and delivery of successful outcomes ^31^. 

This paper is the third in a series of papers which explore enablers and barriers to team interventions. Previous papers focused on the development of initial programme theories (IPTs) through a systematic search of the literature using realist synthesis ^32^ and interviews with key informants ^28^. These IPTs describe the conditions in which multi-disciplinary team interventions appear to work best and why team interventions work best in these conditions

The next phase of the research will elaborate on previous findings by testing these previously developed IPTs in two diverse acute hospital contexts. Findings will therefore be novel. This paper sets out the protocol for this phase of the research.

## Methods

### Context - realist evaluation

Having explored use of realist evaluation in studies relating to complex interventions in healthcare ^7, 33, 34^, realist evaluation ^35^ was considered an appropriate methodology to explore enablers and barriers to team interventions in acute hospital contexts. As a theory based evaluation, realist evaluations aim to unpack “what works, for whom, under what conditions, why, to what extent and how, using Context-Mechanism-Outcome Configurations (CMOCs) as units of analysis. ^7^. Please refer to Table 1 below for an explanation of realist terminology used in this protocol paper.

**Figure 1.**
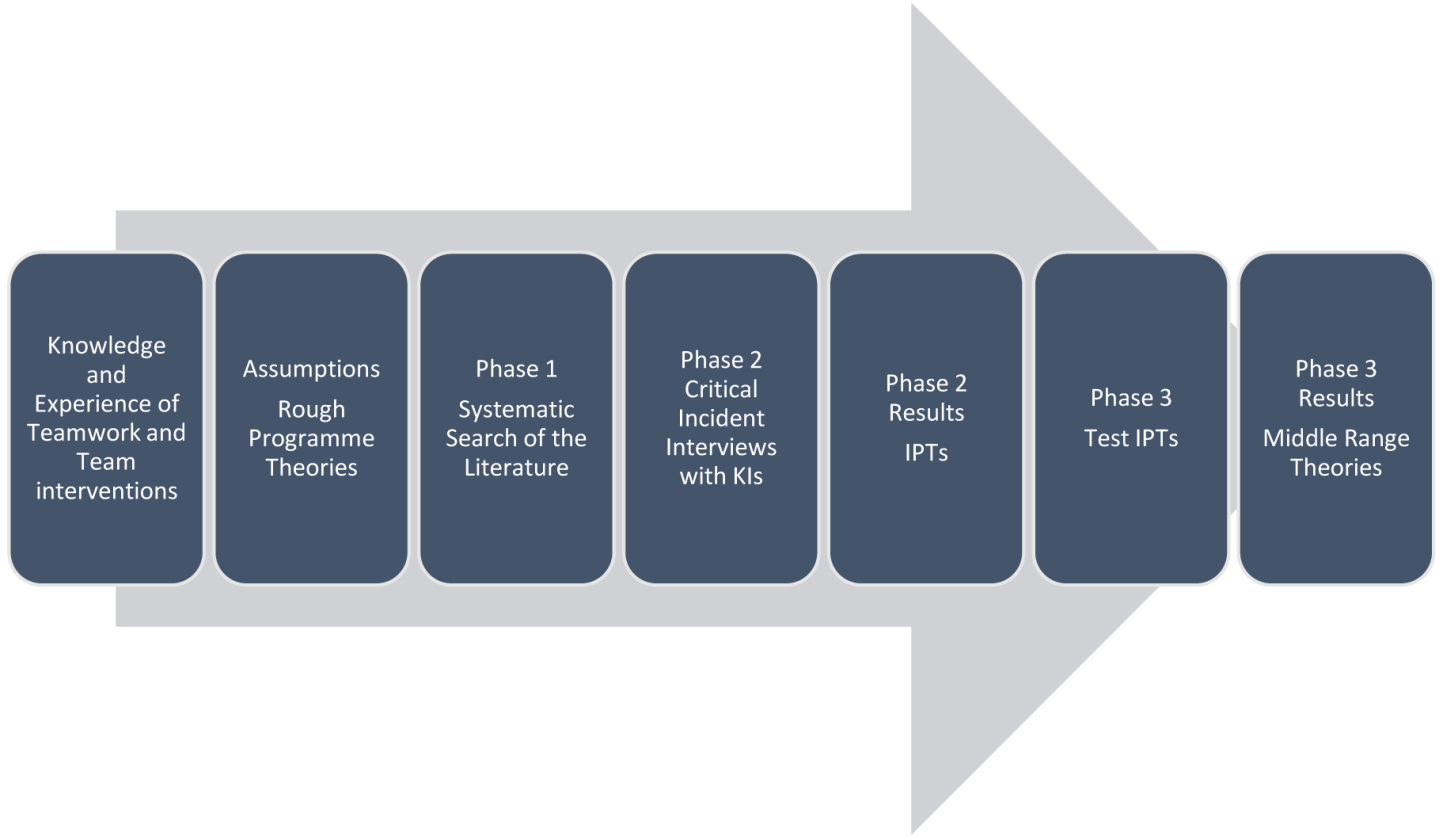
Framework for realist evaluation.

Figure 1 below depicts an overview of the realist evaluation framework adopted for this research.

**Table 1.** Realist terminology.

CMOC	Definition
Context	Those features of the situation into which programmes are introduced that affect the operation of programme mechanisms ^36^.
Mechanism	A combination of resources offered and the participants reasoning in response
Outcome	The intended and un-intended consequences of the intervention.
Configuration	Context-Mechanism-Outcome-Configuration (CMOC) - Patterns and variations in patterns
Demi-regularity	Semi-predictable pattern of occurrences within the data
Initial Programme Theory	The programme architect’s articulation of how the intervention is expected to lead to its effects and in which conditions it should do so
Middle Range Theory	“Theories that have a common thread running through them traceable to more abstract analytic frameworks” ^35^. p. 123

As outlined in Figure 1, this research involves three phases. Phase 1 and phase 2 have already been completed and are reported in detail elsewhere ^28, 32^. These are summarised below in order to provide background context for phase 3.

***Phase 1 - systematic search of the literature***

The first phase of the realist evaluation ^32^ involved a systematic search of the literature using realist synthesis. Consistent with the realist evaluation approach, this was driven by the primary researcher’s (UC) own knowledge and experience of team interventions in an acute hospital context. The primary researcher’s assumptions led to rough programme theories which formed the basis for the search strategy for the review. Relevant literature on team interventions in acute hospital contexts was explored via systematic search processes to determine what worked for whom in what conditions, why to what extent and how. Using realist synthesis, five plausible hypotheses were identified and presented in the form of context, mechanism, and outcome configurations (CMOCs) as per Table 2 below ^32^.

**Table 2.** Five plausible hypotheses from systematic search of the literature using realist synthesis.

	Context	Mechanism	Outcome
* If there is: this enacts* : *and results in:*			
*PH1*	Inter-disciplinary focus and Flattened hierarchy	Understanding of roles & Mutual respect, support and value Shared decision making and common purpose; self and team efficacy	Increased job satisfaction, higher performance, higher levels of competence, and better teamwork and lower feelings of emotional exhaustion. Breaking down of inter-professional silos; more integrated patient care; connectivity of the team and camaraderie.
*PH2*	Effective Communication: *Opportunities for communication; Communication skills;* *Communication systems*	Shared mental models; Clarity of role; Clarity of purpose	Situational awareness; More integrated care; Better intervention outcomes;
*PH3*	Leadership Support & Alignment of team goals with organisational goals	Motivates, empowers and engages staff, creating a sense of team efficacy and a shared sense of responsibility and accountability	Team pride; Camaraderie; Connectedness with broader system; Implementation of Intervention; Sustainability of intervention
*PH4*	Credibility of intervention *provided by experienced trainers who team members can* *relate to and is perceived to be comprehensive (right amount* *of core topics) with application to the healthcare context in* *which the team works,*	A sense of confidence and engages and motivates team members with the intervention	High satisfaction; Increased skills, Increased self and team efficacy, Increased role in safety and translation to practice.
*PH5*	Team composition & Physician involvement - consists of appropriately skilled members including a physician, shares a similar foundational knowledge prior to the intervention and participates in a shared learning experience	Shared understanding of the intervention and feel knowledgeable, competent and confident resulting in	Credibility of the intervention, translation to practice and sustainability.

This table has been reproduced with permission from the authors ^32^.

***Phase 2 - critical incident interviews*** (Use of key informants to refine plausible hypotheses)

Phase 2 of the research involved building of the IPTs by seeking the views of key informants (KIs) (hospital workers directly involved in the design or delivery of team interventions) on the plausible hypotheses which had been developed in Phase 1. Flanagan’s Critical Incident Technique was adapted to seek the views of 17 KIs who were asked to recall both a positive and negative experience of a team intervention with carefully selected probes used to seek their views on the 5 plausible hypotheses. Adhering to RAMESES guidelines ^37^ for realist evaluation, data were analysed using a retroductive approach ^38^ from a total of 31 incidents. The plausible hypotheses were refined iteratively via a series of consultation sessions between the primary researcher and research team with a methodology expert panel ^i^.

This phase of the research resulted in the production of seven IPTs outlined below in Table 3.

**Table 3.** Initial programme theories.

CMOC	Context	+ Mechanism	= Outcome
1* Inter-disciplinary team approach and Flattened hierarchy	*If* Each team member’s voice is heard and considered of equal value	*Then this enacts:* Understanding of roles, mutual respect, support and value Self & team efficacy Perception of shared decision making Common purpose	*resulting in:* Increased job satisfaction Higher levels of competence Better teamwork Lower feelings of emotional exhaustion Breaking down of inter-professional silos More integrated care Connectivity of the team and Camaraderie and More efficient use of time
2* Effective Communication and Shared Understanding of Goals	*If* There is clear, simple, open, honest and timely communication in an appropriate and inclusive environment with SMART goal setting	* Then this enacts:* Shared understanding and clarity of role and purpose; Self- worth and value; Perceptions of confidence and trust in the Intervention	* resulting in:* Positive engagement of the team Situational awareness More integrated planning More efficient use of time and Better chance of success
3* Leadership support and alignment of team goals with organisational goals	*If* There is genuine leadership support in the form of tangible resources and positive acknowledgement of staff and alignment of team goals with organisational goals through effective engagement and dialogue	* Then this:* Motivates, empowers and engages staff, Enacts a sense of team efficacy; a perception of sense making and a shared sense of responsibility and accountability	* resulting in:* Team pride and camaraderie; Connectedness and confidence in the broader system; Easier implementation and sustainability of the intervention
4 Characteristics of intervention that give credibility	*If* The intervention is facilitated/ driven by experienced facilitators who staff can relate to and trust With appropriate clinician involvement where relevant And has perceived relevance to practice with clearly defined goals/outcomes	* Then this enacts:* Team pride and camaraderie; Connectedness and confidence in the broader system; Easier implementation and sustainability of the intervention	* resulting in:* Team pride and camaraderie; Connectedness and confidence in the broader system; Easier implementation and sustainability of the intervention
4a Evidence , recognition and celebration of success	*If* there is evidence of a positive outcome and When there is recognition and acknowledgement that an intervention is successful	* Then this:* Empowers motivates and incentivises staff	* resulting in:* Externally perceived credibility in the intervention and subsequent *buy in* With increased likelihood of further engagement and spread of the intervention and/or future team interventions
5* Appropriate Team composition and Physician engagement and support	*If* there is broad and purposeful selection of team composition *with* • Physician engagement and support if intervention has a clinical focus	* Then this enacts:* Feelings of knowledge confidence and competency Psychological safety and Perception of power and influence	* resulting in:* Legitimacy of the Intervention Better and more timely “buy in” Staff satisfaction Translation of intervention outcomes to practice and better chance of sustainability
6* Personal Relationships	*If* team members have positive personal relationships or prior experience of a positive working relationship and/or an established social network	* Then this enacts:* Perceptions of Trust Perceptions of Psychological Safety Shared understanding of experiential knowledge of team: ways of working, skill-sets likes and dislikes	* resulting in:* Better engagement in intervention and Easier implementation Ability to progress intervention issues informally Distribution of work according to skill-sets More honest and open communication More integrated planning Quicker recovery from conflicts
7 Inter-professional tensions	*If* there are inter-professional tensions, rivalry and mis -trust	* Then this enacts:* Feelings of frustration; lack of respect; dis- empowerment, perceptions of lack of psychological safety and cynicism	* resulting in:* Failure to progress the intervention, lack of support for the intervention and/or withdrawal from the process
7a Escalating mechanisms	*If* There is failure to progress an intervention, lack of support for the intervention and/or withdrawal from the process because of inter- professional tensions	* Then this enacts:* further escalating mechanisms of dis-satisfaction, depletion of energy and resilience and perception of powerlessness	* resulting in:* Greater silo mentality among professions

This table has been re-produced with permission from the authors ^28^

The next phase of this research (Phase 3) will involve testing of theses IPTs. 

***Phase 3 - testing IPTs***

*Ranking exercise*

The seven IPTs developed via phase 1 and phase 2 of the research were first presented to a content expert advisory panel for discussion and refinement. Please refer to Table 4 below for further information on composition and expertise of this content expert advisory panel.

**Table 4.** Composition and expertise of content expert advisory panel that participated in theory ranking activity – (Phase 3).

Content Expert Advisory Panel	
*Composition* 2 Professors in Quality and Safety and Leading international experts on teamwork National Health Service Senior Manager Hospital Group CEO Hospital Group Director of Human Resources National experts in teamwork Patient Advocates	**Descriptor** Experts on teamwork subject matter and quality and safety in healthcare. Senior Healthcare Managers with operational expertise in acute hospital contexts. Individuals who are currently conducting research in the Irish healthcare context or are renowned for their experiential knowledge in the subject. Service users with knowledge of acute hospital contexts from a user’s perspective

Following a brief presentation and discussion of the 7 IPTs with the content expert advisory panel, a ranking exercise was undertaken to enable them to prioritise five of the seven IPTs for testing. The panel ranked the theories in terms of importance on a scale of one to five to reduce the IPTs to a manageable number for evaluation purposes. Two of the seven IPTs were thus eliminated from testing: *IPT 4 Characteristics of intervention that give credibility* (and its corresponding ripple theory *IPT 4a Recognition and celebration of success)* and *IPT 7 Inter-professional tensions (and corresponding IPT 7a Escalating mechanisms)*. The content expert advisory panel perceived the other IPTs to be of more importance for testing, citing various reasons including for example relevance to practice and degree of existing evidence for theories. 

Therefore, the five IPTs chosen for testing (as indicated with an * in Table 3) were as follows:

IPT 1 Interdisciplinary team approach and flattened hierarchy
IPT 2 Effective communication and shared understanding of goals
IPT 3 Leadership support and alignment of team goals with organisational goals
IPT 5 Appropriate team composition and physician engagement and support
IPT 6 Personal relationships

*Testing IPTs via case studies*

In order to develop an in-depth understanding of how and why contexts interacting with mechanisms produce the intended and/or unintended outcomes in team interventions, it was agreed with both advisory panels that these five IPTs should be tested in two different acute hospital contexts using two different team interventions. This will result in further refinement of the initial programme theories in order to progress towards development of middle-range theories (MRTs) that are more widely generalisable.

As per Pawson, workplace interventions are ‘active’ rather than “passive programmes” continuously responding to contextual factors and emerging processes ^39^. Exploring two different team interventions in two different contexts will allow for a broader array of factors, thus providing more rigorous testing of the IPTs. Conditions in one case may enable some mechanisms and consequently trigger intended outcomes whilst contextual conditions have potential to impact these mechanisms differently in the second case resulting in different outcomes. In essence, this will determine how the theories “hold up” within and across both contexts and will yield information that indicates why teams under certain conditions work (generative mechanism ) and the conditions that are needed for a particular mechanism to work (specification of contexts) ^35^.

It will be important to first understand how the team members respond to the respective intervention in each of the two case contexts in terms of their reasoning and the subsequent behavioural change that occurs. ^27, 28^. The team intervention *and* the conditions within which the intervention was implemented will have determined the outcomes of the intervention ^40^. Team members within the same context may have different understanding of contextual conditions in that context, for example the impetus for change or detail of the specific intervention process. Their individual interaction and reasoning with these different contextual conditions therefore needs to be understood in terms of “generative causality” i.e. from their perspective *how and why* outcomes came about.

The underlying social and psychological drivers that drive both intended and unintended intervention outcomes for team members in each of the two different contexts will therefore first be unpacked in the form of CMOCs. Patterns of regularity will be extrapolated from across team member interviews within each case and will be evaluated to discern whether they support, refute or require further refinement of the initial programme theories.

The refined programme theories (again in the form of CMOCs) from each of the individual cases will then be synthesised in terms of their usefulness and efficacy *across* both cases. The CMOCs will thus iterate backwards and forwards in this process of refinement towards development of a middle range theory (MRT):

“Theories that have a common thread running through them traceable to more abstract analytic frameworks” ^35^. p. 123 and “are close enough to observed data to be incorporated in propositions that permit empirical testing” ^35^. p. 22

Moving CMOCs from initial programme theories to refined programme theories and subsequently to middle range theories in this iterative process will allow for the development of a generic set of principles that will be broadly transferable to other acute hospital contexts. The MRTs will provide valuable information to support design, facilitation and implementation of team interventions in acute hospital contexts.

### Case study selection

For the most rigorous testing, together with the content and methodology expert advisory panels, the primary researcher and research team agreed to the choice of two different team interventions from two different hospitals, operating in two different health systems, one in Ireland and one in the USA. 

Table 5 below includes descriptors of the two cases.

**Table 5.** Case study contexts.

Criteria	Case Study 1	Case Study 2
Health system context	Ireland	The Pacific Northwest of the United States
Hospital Type	Quaternary academic public teaching hospital ( >600 beds) All staff are employed by the hospital.	Quaternary academic not-for-profit medical centre ( > 450 beds) Staff are employed by the hospital with the exception of physicians who contract with the hospital.
Intervention descriptor	To change the process for daily general internal medicine (GIM) *takeover of* *care* from the daily *post- call round*.	To strengthen inter-professional collaborative practice and facilitate practice transformation through development and implementation of structured inter-professional bedside rounds (SIBR).
Primary goal	To ensure care of patients admitted from the Emergency Dept. via the un-scheduled care medical pathway is taken over by the most appropriate medical specialty within 24 hours of admission where possible and that there is a more equitable daily distribution of workloads across all medical specialties to ensure safer and better quality of patient care.	To improve relational co-ordination (team communication and relationships) because of high Registered Nurses (RN) turnover; low patient satisfaction and high re-admission rates for patients.
Intervention driver	Internal- Division of Medicine and hospital management	Internal and external- An academic practice partnership between the School of Nursing and the AHF care team with funding from the Health Services Resources Administration (HRSA).
Leadership Support for the intervention	Leadership support included active participation and attendance of the Chief Executive Officer (CEO), Chief Operations Officer (COO) and Executive Clinical Director (ECD) at all meetings and workshops related to the intervention.	Leadership support included attendance during project initiation and close out and at a celebratory workshop.
Team Structure and Composition	The intervention involved formation of a “GIM project team” Following an invitation from facilitators to the GIM group for representation from each medical specialty, medical consultants self -selected to participate in the project team. The CEO, COO and ECD were considered core team members and the team was facilitated by internal facilitators with expertise in lean methodology and organisational change.	The intervention involved formation of a “change team” comprised of inter-professional front-line care team members and grant team members. This purposefully selected change management team comprising multiple disciplines (medical nursing and allied health professionals) from across the advanced heart failure (AHF) faculty and was facilitated by an external research team.
Duration of Intervention	15 months	5 Years
Methodology	The team intervention was underpinned by lean six sigma *Define Measure* *Analyse Improve Control (DMAIC)* methodology. An intensive data collection phase was followed by a workshop to co-design a new way of working and was subsequently followed by a series of monthly meetings and workshops interspersed with smaller stakeholder engagement sessions. A new process was trialled and iterated over three *Plan Do Study Act* cycles. This was followed by a six month control phase once the intervention was embedded.	Following a grant application process and formation of the change team, one year intensive training in TeamSTEPPs was followed by a longitudinal series of twelve leadership workshops delivered over a three year period on a quarterly basis. Workshop content was tailored to needs identified by the change team and incorporated a shift to Structured Inter- professional Bedside Rounds (SIBR) in the in-patient setting. Purposefully selected workshop topics included: improving work and team processes; communication; relational co-ordination using a variety of evidence based interventions e.g. TeamSTEPPs, leadership coaching and presentations from field experts
Numbers of interview participants out of total no of team members invited to participate	N= 19/22 (86%)	N= 16/24 (66 %) *Only front-line care team member participants were invited to participate in* *interviews (not members of the research team or TeamSTEPPs trainers)*

Through the analysis and testing of the IPTs in two diverse cases geographic and healthcare contexts, it will be possible to develop a deeper understanding of the contextual enablers and barriers for team interventions at the team level, as well as exploring whether enablers and barriers differ according to the respective national healthcare contexts.

### Case study 1 (Irish context)

***Intervention descriptor and primary goal.*** This team intervention was designed to change the process for daily general internal medicine (GIM) takeover of care from the daily post- call round in an academic teaching hospital context in Ireland. Prior to this team intervention, the practice was that all medical patients were automatically assigned to the care of the “GIM on- call team” for that night and remained under their care with consults requested from other specialties or requests made to take over care if deemed appropriate. The primary goal of the intervention was to ensure patients were cared for by the most appropriate medical specialty for their needs within 24 hours of admission (where possible) and to ensure that there was a more even distribution of workloads across specialties on a daily basis. This intervention was introduced because of a very large caseload for the respective medical specialty on the day post- call and delays in terms of takeover of care and/or in -patient consults from other specialties. These in-efficiencies were resulting in delays with clinical decision making and discharge planning and consequently resulted in protracted lengths of stay for medical patients. Larger caseloads also had potential to impact quality and safety of patient care.

***Sample and recruitment.*** Members of this *“GIM project team”* will be invited to opt-in and to register their consent to participate in the study by the primary researcher (UC) via e-mail correspondence two weeks in advance of scheduled interviews. As the primary researcher was involved in delivering the GIM project, interviews will be conducted by another member of this research team who is an experienced qualitative researcher (EMcA). 

***Data collection.*** Interviews using an interview guide informed by the IPT will be used to collect data from the individual participants in case study 1. During the first part of these one-to-one interviews with the GIM project team members, information will be gathered about the team intervention, the composition of the team, how the team operated and team processes. Subsequently, theory-driven interviews using an adapted form of the Teacher- Learner style interview technique ^34^ will be used to test the initial programme theories that have been informed by the extant literature and the data collected from KIs in Phase 1 and 2 of the research. Interviewees will be invited to comment on theories that are introduced by the interviewer thereby allowing them to confirm or refute them ^34^ and in this way, the IPT will be refined. 

Please refer to extended data for a detailed outline of the interview format ^41^.

Data will be collected at a location and time suitable for participants. All interviews will be audio-recorded. The qualitative data accumulated from these interviews will provide insight into how and why the multi-disciplinary team intervention was enabled or inhibited and give insight into the experiences of those affected by the intervention, as well as the intended and unintended consequences of the intervention ^35^.

### Case study 2 (US context)

***Intervention descriptor and primary goal.*** This team intervention was designed to strengthen inter-professional collaborative practice and facilitate practice transformation through development and implementation of structured interprofessional bedside rounds (SIBR) at a large medical centre in the Pacific Northwest, USA ^42– 44^. The primary goal of the intervention was to improve healthcare team, healthcare system, and patient outcomes for hospitalised patients with heart failure with particular emphasis on relational co-ordination (team communication and relationships) because of high staff turnover, low patient satisfaction and high re-admission rates for patients.

***Sample and recruitment.*** This case was identified by the authors as meeting research criteria and constituting a suitable team intervention in a contrasting context that will enable further testing of the initial programme theories. Once the appropriate members of the US research team were identified, an overview of the research including: the research question, the methodology and the IPT was given via a power point presentation by the primary researcher (UC). The goal of the secondary analysis proposed was explained in detail i.e. to examine the stability of the IPTs in terms of what enabled and/or inhibited the multi-disciplinary healthcare team intervention using the US case which differed in terms of health care context, team composition and intervention detail. Following a comprehensive discussion, it was agreed that the data from interview narratives (n= 16) conducted ^31^ with the change team upon completion of the intervention would be suitable for this purpose.

***Data transfer.*** Data from the 16 interview narratives for case study 2 will be transferred as per a data sharing agreement. Confidential information will be protected through encryption.

Please refer to extended data ^41^ for a detailed outline of the interview format that was used in the primary study and which has been reported elsewhere ^43^. 

### Organisational contexts case studies 1 and 2

Prior to commencing the research study, the researcher will develop an understanding of the broader hospital context in each case at the time of the intervention being completed. This will be done both reflexively by reviewing relevant documentation for example, relevant publications, and minutes of meetings or e-mail correspondence relating to the intervention and more pragmatically by developing field notes from meetings with appropriately identified staff. Details of the drivers of the intervention and how they aligned with the overall hospital’s strategic plan, quality and safety agenda and key performance indicators will be sought.

Relevant data sets relating to the intervention, for example quantitative data relating to intervention impacts will be reviewed as required. Depending on how the evaluation evolves and requirement for deeper insights relating to the generative causation, further meetings may be scheduled to clarify specific pieces of information.

### Data analysis case studies 1 and 2

Data analysis and synthesis will be informed by Gilmore *et al.*’s guidelines for data analysis and synthesis within realist evaluation *(Phases 3–5)* which are outlined in Table 6 below ^45^. It is expected that all data will be extracted and analysed by June 2021.

**Table 6.** Data analysis and synthesis.

Data analysis and synthesis within realist evaluation ^45^	
Phase 3	Step 1 Data preparation Step 2 CMOC * extraction and elicitation
Phase 4	Step 1 Using CMOCs * to refine IPTs Step 2 Collating evidence and refinement verification
Phase 5	Step 1 Synthesis across studies for MRTs

*CMOC- Context-Mechanism-Outcome-Configuration.

***Data preparation.*** Data from the audio files will be transcribed *(CS1)* and uploaded *(CS1 and CS2)* to NVivo 12 software ^46^. Transcripts will be read and initial observations and annotations made. 

***CMOC extraction and elicitation.*** CMOCs will be used as the units of analysis. As per realist evaluation, best practice guidelines ^37^, using deductive reasoning and inductive reasoning - CMOCs will be extracted and/or new CMOCs will be elicited from the interview narratives and coded to corresponding NVivo nodes that reflect the 5 IPTs or newly created nodes for additional CMOCs elicited.

***Using CMOCs to refine IPTs.*** Using deductive reasoning ^45, 47, 48^, the IPTs will be tested to determine whether the perspectives and account of interviewees support or refute the IPT. In addition, via a process of inductive reasoning ^45, 47, 49^, new information may result in refinement of the existing theories and the development of further new theories if a series of observations are made and new patterns of regularity emerge in terms of generative causation of outcomes and un-intended outcomes.

***Collating evidence and refinement verification.*** A retroductive approach i.e. a process of moving backwards and forwards between the data within each case searching for clarification of support, refute or refinement will be used to determine how the CMOCs align with the original IPTs. All decisions and thought processes will be logged in linked memos for the purposes of transparency.

In order to ensure rigour and robustness of the process, a random sample of four narratives from each case study will be double coded by another member of the research team and co-author (ADB).

***Synthesis across studies for MRTs.*** Following data analysis within cases, data analysis will then move to synthesis and refinement of theories across cases in order to reach middle range theories. This will be informed by the results of data analysis within each respective case study and will incorporate a search for demi-regularities (semi predictable patterns occurring in the data) across the two case studies.

As the evaluation progresses, the methodology expert advisory panel may be contacted with regard to data analysis in order to assist and challenge decision making and in so doing, to optimise quality of research design and methodological rigour. The evaluation therefore will not progress in a linear fashion.

Further engagement with the content expert advisory panel may also be warranted as well as refinement based on focussed reviews of relevant literature. This iterative process of seeking advice at each stage from the expert advisory panel is a recommendation from the RAMESES guidelines for realist evaluation ^37^.

## Ethics and dissemination

Favourable ethical opinion has been received from University College Dublin Ethics Committee (HREC-LS-16-116397) for this research without requirement for further ethical review (LS-E-19-109) for testing in external contexts. Written permission was secured from the organisation involved in the first case study and recruitment of participants and other data collection did not begin until this was in place. Human subject’s approval from the US-based institution was not needed given that the initial study was deemed exempt from the Human Subjects Review Board and only de-identified data were to be transferred. A data sharing agreement between the authors and the research team from the US academic institution was subsequently drawn up, agreed and signed by both parties. De-identified transcripts were not shared until this was in place.

In accordance with University College Dublin’s policy on data protection and storage, any paper versions of notes will be anonymised and will be stored securely and only accessible to the members of the research team.

Results will be disseminated via peer-review journals, national and international conferences and presentations to relevant stakeholders and interest groups for example: quality and safety governance groups, clinical audit and effectiveness committees and HSE Quality and Information Division (Ireland) and US research fora as deemed appropriate by the US research team. The findings will also be published in peer review journals.

## Study status

All participants have been contacted and consent has been obtained for case study 1. Ethical approval has been obtained. Data for case study 2 has been anonymised and prepared for transfer for the purposes of secondary analysis. Ethical approval has been obtained to support this secondary analysis.

## Discussion

Understanding the contextual conditions under which team interventions are undertaken and how these contextual conditions interact with team members’ reasoning as individuals and as a collective will be helpful in order to understand how and why implementation of some interventions might fail or flourish. Realist evaluation is a complex research design and allows deep exploration and insights to be developed which consider the influence of contextual factors when exploring the enablers and barriers to multi-disciplinary team interventions in acute hospital contexts. 

This work is novel as unlike other research which focuses on whether interventions work or not, it will explore how and why interventions work, what specific contextual and team factors enable team members’ as individuals and as a collective to work effectively to produce successful outcomes. Given the importance of teamwork to delivering healthcare, a better understanding of these factors will be valuable for education, training and development of hospital teams.

Realist evaluation is theory driven and is in keeping with an interpretative process. It seeks to deepen understanding of ‘what works, for whom, in what conditions, why, to what extent and how, as opposed to more traditional empirical studies, which look for more definitive answers of whether an intervention works or not ^7^. Examining different contexts by using realist evaluation allows for a more rounded comprehensive approach and takes into account a broader range of perspectives.

Realist evaluation is being employed for this research because it is innovative and insightful and will allow deconstruction of the causal web of conditions underlying team interventions whilst grounding it in the ‘ *messy reality’* of healthcare. A realist evaluation yields information that indicates how the intervention works and the conditions that are needed for a particular mechanism to work and, thus, it is likely to be more useful than other types of evaluation in making recommendations for the design of team interventions.

Due to the variation in the contexts in which multi-disciplinary healthcare teams operate and the teamwork mechanisms enacted in those contexts, there may be many different outcomes from team interventions. Realism is not obsessed with the target of a single pass or fail outcome. Instead, use of this methodology to test the IPTs will enable a more sensitive look at patterns and variations in patterns of multi-disciplinary team interventions, for example:

▪ The conditions in which team interventions are introduced- the enablers and barriers to success of these interventions
▪ How the resources on offer permeate into the reasoning of team intervention participants
▪ The intended and un-intended consequences of team interventions
▪ How any one of the components of team interventions brings about change
▪ Why team interventions work under certain circumstances

This research will therefore have a practical application for educators, managers, policy makers and decision makers in terms of providing recommendations on how to enable team effectiveness when delivering team interventions and thereby improve quality and safety in delivery of care for patients. This will help to ensure its relevance and application in ultimately improving team performance and enhancing patient safety cultures.

Outputs of this work will be applied directly to the implementation of interventions to improve team working in acute hospitals, will inform local work in healthcare transformation as well as influencing work on development of multi-disciplinary team interventions in the national and international context.

As the IPTs were informed by hospital workers directly involved in the design or delivery of team interventions and will be tested using case studies from two different hospital systems, this ensures that the middle range theory reached will be grounded in the reality of everyday experiences of hospital staff. Use of data from interviews in the two case studies will enable a comprehensive assessment of the team intervention from several perspectives. By understanding the contextual factors and the mechanisms through which outcomes are mediated, realist evaluators conclude that findings and recommendations are therefore more relevant ^37^. 

This phase of testing the IPTs will help to further topic development in terms of understanding how and why multi-disciplinary team interventions in acute hospital contexts are impacted by various contextual conditions in terms of generating specific outcomes. The engagement of hospital staff and expert advisory methodology and content panels consisting of senior academics, patient representatives and senior hospital managers as well as the researchers will help to ensure robustness, relevance and rigour of the research.

There is a diverse group of institutional partners involved in this research and it is intended in addition to peer reviewed publications that each research partner will utilise their existing networks and partnerships to discuss and disseminate findings. The influence and impact of this study will thereby extend beyond a single context. 

## Patient and public involvement

Two patient advocates were involved in the ranking of initial programme theories for testing as part of the content expert advisory panel.

## Reporting guidelines

RAMESES II reporting standards for realist evaluations ^50^ will be adhered to for reporting purposes of this study.

## Data availability

### Underlying data

No underlying data are associated with this article.

### Extended data

Dryad: Appendices interview formats. https://doi.org/10.5061/dryad.q83bk3jg8 ^41^.

This project contains the following extended data:

- Appendix_1_Interview_format_Case_Study_1-2.docx
- Appendix_2_Interview_format_Case_study_2-2.docx

Data are available under the terms of the Creative Commons Zero "No rights reserved" data waiver (CC0 1.0 Public domain dedication).

## Footnotes

^i^an interdisciplinary group of researchers and academics with a specific interest in, and experience in applying, realist methods
