# Supplementary material for: Team interventions in acute hospital contexts: protocol for the evaluation of an initial programme theory using realist methods
Source: HRB Open Res. 2021 Mar 29;4:32. [Version 1] doi: 10.12688/hrbopenres.13225.1 (PMC9051586; doi:10.12688/hrbopenres.13225.1)
Supplement: Peer review history

# Peer review: Reviewer response for version 1

**DOI:** 10.21956/hrbopenres.14383.r31862

**Published:** 2022-04-28

**Authors:** Bronagh Blackwood

**Affiliations:** 1 The Wellcome-Wolfson Centre for Experimental Medicine, Queen's University Belfast, Belfast, UK

This research is highly relevant considering the continuing interest in healthcare research in the evaluation of complex interventions: particularly those that involve a behaviour change from an established practice to adoption of a new approach. In such cases, the gold standard randomised controlled trial alone provides insufficient evidence to explain *why* a team-based intervention does or does not work. Thus this approach, that places the emphasis on evaluating the context into which a team is based and the mechanisms of their interactions, is highly important.

 The protocol is well written, clear and robust in its presentation of the plan to conduct a realist evaluation of two team-based interventions. The authors present a clear overview of the literature and a logical justification for the need to approach the evaluation of team-based interventions using this method. The chosen case studies represent two different interventions in different contexts and in two countries with very different healthcare organisations and resources. This is a strength in that evaluation of the initial programme theories is being tested more broadly and the findings may be more relevant for developing a mid-range theory. Had the authors been able to sample an additional case study from a lower income country, it could have been more interesting and broadly applicable. Perhaps something to be taken forward in future research.

Is the study design appropriate for the research question?

Yes

Is the rationale for, and objectives of, the study clearly described?

Yes

Are sufficient details of the methods provided to allow replication by others?

Yes

Are the datasets clearly presented in a useable and accessible format?

Yes

Reviewer Expertise:

Critical care research, clinical trials and evaluation of complex multicomponent interventions requiring practice change. Methodological expertise in realist evaluation and process evaluation.

I confirm that I have read this submission and believe that I have an appropriate level of expertise to confirm that it is of an acceptable scientific standard.

# Peer review: Reviewer response for version 1

**DOI:** 10.21956/hrbopenres.14383.r29427

**Published:** 2021-05-24

**Authors:** Lesley Middleton

**Affiliations:** 1 Health Services Research Centre, Victoria University of Wellington, Wellington, New Zealand

This realist-based research addresses the challenge of providing a more sensitive look at the patterns and variations in patterns of multidisciplinary team interventions. As the protocol notes, what constitutes a multidisciplinary team intervention can vary, but overall, there is a body of literature highlighting the importance of strong teamwork as an important component in successful quality improvement interventions. The design of the study is appropriate for the questions being asked. 

 A review of the final five IPTs that the study will be testing, reveals a wide diversity of outcomes. These diverse outcomes range from those linked to; individuals and their increased job satisfaction, teams who collectively show pride and camaraderie, patients who receive improved integration, and management who experience better translation of change ideas into practice. The claim that “MDTs are the vehicle for improvement in quality and safety” is a confusing as there can be other names (i.e. learning collaboratives) and other actions (i.e. change workshops) that can be understood as “vehicles” for an quality improvement intervention.

 The MDTs under investigation are: Case study 1, a new GIM project team, and Case study 2, structured interprofessional bedside rounds. Case study 1 was hard to follow as most of the description focused on what the process replaced rather than what it did. It would be useful to know more about the ways this was a change relying on multidisciplinary teamwork. 

 Following the realist logic of inquiry, commendable focus is given to developing initial programme theories to test through data gathering and refining. A content advisory panel ranked IPTs developed from an earlier realist synthesis on a scale of 1-5. The assumption is that the final IPTs were judged by the panel as having stronger explanatory power linked to a critical incident experience.

 The authors have well described their planned analysis and search for CMOCs to refine their IPTs. There will be some challenges as Case Study 2 involves a secondary analysis of interviews conducted less in a realist teacher learner style. The result is that the raw interview data may contain more descriptions than explanations. The authors have built in iterative processes to further refine their theories. This should allow them to address any inconsistencies that arise between datasets.

Is the study design appropriate for the research question?

Yes

Is the rationale for, and objectives of, the study clearly described?

Yes

Are sufficient details of the methods provided to allow replication by others?

Partly

Are the datasets clearly presented in a useable and accessible format?

Yes

Reviewer Expertise:

Realist research, health services evaluation, healthcare policy

I confirm that I have read this submission and believe that I have an appropriate level of expertise to confirm that it is of an acceptable scientific standard.
